# The Oxygen Mixture as an Anæsthetic

**Published:** 1869-05

**Authors:** E. Andrews

**Affiliations:** Prof. of Principles and Practice of Surgery in Chicago Medical College; Chicago, No. 81 Monroe St.


ARTICLE XX.

### THE OXYGEN MIXTURE AS AN ANÆSTHETIC.

By E. ANDREWS, M.D., Prof. of Principles and Practice of Surgery in Chicago Medical College.

Some months since I gave in the *Examiner* an account of my experiments in the use of a mixture of free oxygen and laughing-gas as an anæsthetic. Special circumstances interrupted for a time my investigations, but the following recent case will serve as an additional example of the action of the mixture:—

Mrs. W. had a fatty tumor on the back of her neck, which she wished removed at her residence. The mixed gas was accordingly carried there in a rubber sac and administered by Dr. Rogers. The inhaler was a silver mouth-piece inserted between the lips, and spreading between the lips and teeth so as to allow of no admixture of atmospheric air. Valves allowed the inspired gas to come from the sac, and the expired air to pass out of an opening into the atmosphere. Thus being prepared, the inhalation commenced. In seventy-five seconds the patient was anæsthetized, presenting a sort of pallor of the lips and slight blueness of the veins, but none of the dark asphyxiated flush and labored breathing produced by unmixed laughing-gas. I proceeded to extirpate the tumor, occupying two minutes in the operation, which I accomplished without any consciousness on the part of the patient. The inhaler was then removed from the mouth, and the patient recovered her senses in about two minutes, waking up pleasantly, without nausea or discomfort. The blood from the incision was a little darker in tint than usual, showing that there was not quite free oxygen enough in the mixture.

*Chicago, No. 81 Monroe St.*

